#  Evidence-Based Options for Controlling Respiratory Virus Transmission

**DOI:** 10.3201/eid2311.171231

**Published:** 2017-11

**Authors:** Benjamin J. Cowling, Tommy Tsan-Yuk Lam, Hui-Ling Yen, Leo L.M. Poon, Malik Peiris

**Affiliations:** University of Hong Kong, Hong Kong Special Administrative Region, China

**Keywords:** Respiratory viruses, transmission, control, aerosols, experimental animal models, nonpharmaceutical interventions, evidence-based, viruses, respiratory infections

Given the speed with which viruses transmitted by the respiratory route spread globally, epidemics caused by these viruses pose great threats to global public health. Recent examples include the global outbreak of severe acute respiratory syndrome caused by a coronavirus in 2003–04 (*1*), the rapid global spread of pandemic influenza A(H1N1) in 2009 (*2*), and the ongoing outbreaks of Middle Eastern respiratory syndrome (MERS) caused by another coronavirus (*3*). Human respiratory viruses such as influenza virus, respiratory syncytial virus (RSV), parainfluenza, adenovirus, and rhinovirus cause considerable illness each year (*4*,*5*). Surprisingly, little is known about the mechanisms by which these viruses are transmitted; much of what is believed to be known is based on dogma. Such knowledge gaps include the relative importance of contact, fomite, and airborne (large droplet versus small droplet) spread; how environmental factors affect different modes of transmission; the aerobiology of virus transmission; the role of viral “quasi-species” and fitness landscape in transmission (*6*); and viral and host determinants of adaptation of animal viruses for transmission in humans (*7*,*8*). In turn, these knowledge gaps compromise the effect and rational use of nonpharmaceutical interventions for infection prevention and control. Such nonpharmaceutical interventions may be the only available options for control of a newly emerged respiratory pathogen such as the severe acute respiratory syndrome coronavirus.

As part of its 130th anniversary celebration, the Li Ka Shing Faculty of Medicine of the University of Hong Kong has provided financial support for several international conferences in its priority research areas, including respiratory virus transmission and control. An international conference on this topic was held June 19–21, 2017, in Hong Kong, attended by >190 participants from 19 countries (http://transmission2017.med.hku.hk/). The Croucher Foundation, an independent private Hong Kong foundation, also provided financial support for this conference.

## Objectives 

Ongoing work addressing some of the gaps in knowledge about respiratory virus transmission tends to occur within isolated individual laboratories, by pathogen (e.g., influenza, RSV, rhinovirus, measles, MERS), or by experimental approach (e.g., those working with experimental animal models, basic virology, epidemiology, evolution, aerobiology, infection control). This conference aimed to cut across these divisions to bring together researchers working with different respiratory viruses and using different experimental approaches.

## Agenda

The conference was organized around 4 themes (Table). An international scientific committee developed the program and invited 21 experts to discuss the full range of the conference themes. Eighty-five abstracts were submitted; 25 were presented orally, and 60 were displayed as posters. The conference presentations discussed transmission of a range of respiratory viruses, including transmission of influenza, RSV, rhinovirus, parainfluenza virus, MERS coronavirus, and morbilliviruses. As an example of a respiratory pathogen about which modes of transmission and control are well understood, available data on transmission of *Mycobacterium tuberculosis* were reviewed.

**Table:** Conference themes, examples of the questions put forward for discussion, and lessons learned at conference on respiratory virus transmission and control, Li Ka Shing Faculty of Medicine of the University of Hong Kong, Hong Kong, 2017

Theme	Examples of questions discussed	Lessons learned
Theme 1: respiratory virus transmission among humans	What proportion of respiratory virus transmission occurs through the aerosol (fine particle) route? How many virions are involved in transmission from person to person for different viruses? How do host, viral, and environmental factors influence the routes of transmission? How much viral evolution occurs at the transmission event, versus during the course of infection within a host? Which locations or environments are more supportive to viral transmission? How do all of these issues vary between viruses?	There is a resurgence of interest in these key questions, with novel methodologies being developed, for example, in air sampling and in investigation of intra-host virus genetic diversity as related to the transmission bottleneck. It was also instructive to learn from the information gained from study of transmission of bacterial infections such as tuberculosis.
Theme 2: animal models for virus transmission	What information can be provided from experimental animal models on viral and host factors associated with enhanced transmission potential and on mechanisms of transmission? What is the effect of different inoculation routes on onward transmission? What is the size range of virus-laden particles that mediate influenza transmission among ferrets? Which animal models have preferable characteristics for different viral infections? What additional parameters may be monitored (e.g., by using air sampling devices) during a transmission study to refine the conclusions?	It was instructive to learn from the animal models used for different viruses. The importance of standardizing how such transmission experiments are carried out is challenging but important for results to be comparable. Use of novel technologies, including new imaging technologies, was discussed.
Theme 3: determinants of transmission	What are the viral and host determinants of the emergence of viruses that are efficiently transmissible in humans? What can we learn from surveillance at the animal–human interface, and how should this be done efficiently? What are the best strategies for risk assessment of the many zoonotic transmission events that occur regularly? How should we choose which infections to prioritize the development of medical countermeasures? Can we refine risk assessment algorithms to identify animal viruses of great zoonotic potential?	The importance of risk assessing data obtained from surveillance was noted. The systematic risk assessment algorithms developed for influenza may serve as a model for risk assessing other emerging respiratory viral pathogens.
Theme 4: control of respiratory virus transmission	Which nonpharmaceutical measures are more effective in reducing the transmission of respiratory viruses, such as source control measures or environmental controls? What is the risk of transmission in different settings and the impact of control measures on risk? What are the best approaches to control nosocomial transmission of respiratory viruses? What is the potential for new strategies to control respiratory virus transmission? How do all these issues vary between viruses? What are the options for prevention of zoonotic emergence?	Improved understanding of airflow and aerosol dispersion in health care settings may lead to improved engineering designs to reduce risks of nosocomial transmission. Improved understanding of transmission of viruses within domestic livestock trading and marketing systems may allow interventions that reduce risk of emergence of novel emerging pathogens.

Presentations provided data on age-specific contact patterns between humans and other animals and their relevance for mathematical modeling of transmission dynamics. Data from large community-based studies on virus transmission were also presented. Such cohort studies may allow defining of a “contagious phenotype.” One theme of interest was the potential for deep sequencing data to permit estimation of the number of viral particles transmitted from one person to another (the transmission bottleneck) and how this bottleneck might vary for different reasons. Other discussions included the potential use of deep sequencing approaches to study virus evolution, viral fitness, and antiviral resistant mutations, as well as the role of environmental factors such as temperature, humidity, and environmental pollutants (e.g., PM2.5) on viral transmission. Social and ecologic factors examined included crowdedness (in human households, in dog shelters) and school holidays.

A novel in vitro experimental approach that allows delivery of aerosolized virus onto human ciliated epithelial cells followed by monitoring changes of physiologic parameters (e.g., ciliary function, mucus production) on these cells was described. There was particular interest in the use of experimental animal models for research on virus transmission, and data were provided on the advantages and disadvantages of different experimental animal models for influenza, RSV, morbilliviruses, and parainfluenza viruses. As an extension of previous work that identified the importance of influenza transmission through respiratory droplets in the ferret model (*9*), a new approach to analyze the particle sizes involved in transmission of influenza in this model was presented. Also discussed were novel imaging technologies and experimental models that allow simultaneous measurements of different viral and host parameters for pathogenesis and immunological studies in vivo.

Another topic was the emergence and maintenance of zoonotic respiratory pathogens, such as avian influenza H5Nx and H7N9, swine influenza H1N1 and H1N2, canine influenza H3N8 and H3N2, and MERS coronaviruses. Risk assessments of these emerging zoonotic pathogens, with particular attention to outbreaks of influenza A(H7N9) in China, were presented. Refining and validating experimental animals and ex vivo and in vitro models for assessing risk of emerging respiratory pathogens remain an area for future research.

A section of the conference focused on the study of airborne virus particles. The performance and advantages of different air samplers were discussed. Data on measurement of virus-laden particles in air in different settings were presented, including experimental animal studies, between humans in community and healthcare settings, and at the animal–human interface, such as live poultry markets in Asia and swine barns and county fairs in the United States.

The final topic in the conference was control of respiratory pathogens. Patterns of aerosols generated by procedures and devices in hospitals were discussed, together with engineering solutions to minimize such risks. Viral and environmental factors that lead to generation of infectious droplets in live poultry markets and slaughtering facilities were investigated, along with options for reducing such risks. The effect of vaccines on reducing virus transmission was also discussed.

## Next Steps

The conference was very well received by delegates, many of whom expressed enthusiasm for the theme and the active interdisciplinary discussions that were a feature of the meeting. There was near unanimous support for this to be the start of a series of meetings with multidisciplinary participation, perhaps at 2–3-year intervals. The International Society for Influenza and Other Respiratory Virus Diseases (ISIRV) provided some logistical support for this conference; a potential development is the creation of a special interest group within ISIRV for conference participants and other researchers to discuss these shared interests.
